# Should We Consider Patients with Coexistent Hepatitis B or C Infection for Orthotopic Heart Transplantation?

**DOI:** 10.1155/2013/748578

**Published:** 2013-11-07

**Authors:** Baskar Sekar, Pippa J. Newton, Simon G. Williams, Steven M. Shaw

**Affiliations:** ^1^The Transplant Centre, Wythenshawe Hospital, University Hospital of South Manchester NHS Foundation Trust, Manchester M23 9LT, UK; ^2^Department of Infectious Diseases, Wythenshawe Hospital, University Hospital of South Manchester NHS Foundation Trust, Manchester M23 9LT, UK

## Abstract

Heart transplantation (HTX) is the gold standard surgical treatment for patients with advanced heart failure. The prevalence of hepatitis B and hepatitis C infection in HTX recipients is over 10%. Despite its increased prevalence, the long-term outcome in this cohort is still not clear. There is a reluctance to place these patients on transplant waiting list given the increased incidence of viral reactivation and chronic liver disease after transplant. The emergence of new antiviral therapies to treat this cohort seems promising but their long-term outcome is yet to be established. The aim of this paper is to review the literature and explore whether it is justifiable to list advanced heart failure patients with coexistent hepatitis B/C infection for HTX.

## 1. Introduction

Heart transplantation (HTX) is the gold standard surgical treatment for patients with advanced heart failure. Worldwide, the median survival in patients who survive the first year after HTX is about 14 years [1]. This has been attributed to advances in immunosuppressive therapy over the years and importantly, the proper selection of patients who will benefit most from this treatment. Patients undergo extensive investigations from routine biochemistry and virology screening to invasive investigations like right heart catheterisation before being considered for heart transplantation. Patients with significant abnormalities from the assessment are deferred treatment. Though previous infection with Cytomegalovirus and Epstein-Barr virus is not an absolute contraindication, reactivation of these viruses after transplant due to immunosuppression can adversely affect long-term outcome [2, 3]. 

Hepatotropic viruses (especially hepatitis B and hepatitis C) affect a large number of people throughout the world and they are one of the commonest causes for chronic liver disease and hepatocellular carcinoma [4]. Worldwide, it is estimated that 2 billion people have been infected with hepatitis B and 150 million with hepatitis C. Around 600,000 and 250,000 individuals die each year of complications associated with hepatitis B and hepatitis C infections, respectively. The prevalence of hepatitis B and hepatitis C infection in HTX population is over 10% [5]. Despite its increased prevalence, the long-term outcome in this particular cohort is still not clear. There is no clear consensus as to whether patients with coexistent hepatitis B or C infection, including individuals with a past history of acute resolved infection, should be considered for HTX. The aim of this paper is to review the literature and explore the suitability and long-term outcome in this cohort after HTX.

## 2. Effect of Hepatotropic Viruses (HBV/HCV) on Graft/Survival Outcome

Cardiac allograft vasculopathy (CAV) plays an important role in predicting long-term outcome in HTX recipients. Several immunological and nonimmunological factors relate to the recipient or the allograft itself are implicated in the pathogenesis of CAV [6, 7]. Of these, viral triggers have been identified to play a significant causative role [2, 8, 9]. The effect of HBV/HCV viruses on CAV/survival outcome has been analysed in a number of studies (Table 1). 

Haji et al. [10] analysed 66 patients with intracoronary ultrasound who underwent HTX between 1998 and 2000. 13 patients were included in HBV group (hepatitis B core antibody is positive in either the donor or the recipient) and 53 patients in the control group (hepatitis B core antibody is negative in both donor and recipient). They found that change in average intimal area and average maximal intimal thickness over a year was markedly increased in the HBV group compared to controls (1.59 ± 1.4 versus 0.46 ± 0.4 mm^2^, *P* = 0.01, and 0.19 ± 0.25 versus 0.07 ± 0.1 mm, *P* = 0.10). The authors concluded that CAV risk is increased when HBV seropositivity is found in either donor or recipient. 

In a multicenter cohort study involving 20,687 HTX recipients, Lee and colleagues [11] assessed survival outcome in 443 HCV-seropositive compared to 20,244 HCV-seronegative patients. During the mean follow-up period of 5.6 years, a significantly higher mortality was observed in the HCV-seropositive group compared to HCV-seronegative group (177 (40%) versus 6,367 (31.5%); *P* = 0.0001). Surprisingly, most of the deaths in the HCV-positive group were due to CAV rather than hepatic decompensation (16.4% versus 3.9%). The authors speculated that increased CAV incidence in this group might be due to immunosuppression worsening chronic HCV-related inflammation. Another possible explanation was the immunosuppression that caused accelerated progression of HCV-associated liver disease.

The other major determinant of long-term outcome in HTX recipients with coexistent hepatotropic viral infections is the development of chronic liver disease. In a retrospective analysis of 360 HTX recipients, Fagiuoli et al. [12] evaluated 49 patients who were tested positive for hepatotropic viruses (HBV 3.1%, HCV 12%, and concomitant infection 0.5%). Over 50% of HCV-positive recipients and all HBV-positive recipients developed chronic liver disease during the follow-up period of an average 8 ± 3.1 years. 16% of them developed cirrhosis and 8% died of end-stage liver disease.

In a retrospective survey of the Joined International Society for Heart and Lung Transplantation/United Network of Organ Sharing (UNOS) thoracic registry, Hosenpud et al. [13] analysed 30 HTX recipients who were tested positive for hepatitis B surface antigen. Active inflammation or cirrhosis was found in approximately 37% as well as a statistically significant relationship between clinical liver disease and hepatitis C antibodies. Though significant difference in survival was not demonstrated compared to the reference HTX group, authors suggested cautious acceptance of HBsAg positive patients for HTX as the majority of deaths were directly related to hepatitis B.

The small number of patients and retrospective nature were a major limitation of these studies. However, increased occurrence of significant liver disease and mortality even in this small cohort is of major concern. 

## 3. Effect of Immunosuppression on HBV/HCV

HTX recipients require a lifelong commitment to taking immunosuppressive drugs (most commonly glucocorticoids, calcineurin inhibitors, and inosine monophosphate dehydrogenase (IMPDH) blockers) to prevent early and late rejection. However, immunosuppressive drugs in HTX patients with coexistent HBV/HCV infections can pose a significant risk as they may reactivate these viruses. When reactivated, a rise in viral replication occurs resulting in a clinical spectrum varying from no symptoms to fulminant liver failure and even death [14]. The effect of immunosuppression on hepatitis B and hepatitis C virus reactivation has been analyzed in a number of studies. 

### 3.1. Calcineurin Inhibitors

Calcineurin inhibitors are the mainstay of immunosuppressive treatment after heart transplantation. Cyclosporin A (CsA), in addition to immunosuppression, has been shown to inhibit HCV replication both in vivo and in vitro [24–27]. In contradistinction, Tacrolimus (FK 506) was found to have no inhibitory effect on HCV RNA replication [26]. Inoue and Yoshiba [27] studied the efficacy of interferon treatment in combination with CsA in 41 liver transplant recipients with end-stage HCV-related disease. The treatment consisted of induction and intensified therapy (interferon beta and CsA) followed by maintenance therapy (interferon alpha 2b and ribavirin). Patients received 200 mg of CsA in four divided doses during the induction and intensified therapy phase (to maintain a trough level of 250–400 ng/mL) in addition to interferon. During follow-up, 65% achieved sustained virological response and 80.4% achieved sustained biochemical response. However, retinopathy developed in 19 patients, severe proteinuria in 5, and encephalopathy in 1 which were felt to be related to CsA necessitating discontinuation in four patients. The authors conclude that better clinical response was achieved when the effect of these two drugs is maximised. 

### 3.2. IMPDH Inhibitors

Mycophenolate mofetil (MMF) is an IMPDH inhibitor with antiproliferative as well as antiviral activity on some viruses including HIV-1, HCV, and HBV [28–30]. The effect of MMF on HBV/HCV has been analysed in a number of studies. In an in vitro study, Ye and colleagues [29] analysed the effect of MMF on HCV replication in human hepatic cells. They found that MMF inhibits HCV replication through depletion of guanosine. In a further study [30], MMF was also found to inhibit HBV replication in vitro. 

Although the above study results appear favourable, the results were not translated well into the clinical studies. In many of the studies, there seem to be a significant viral reactivation and increased incidence of chronic liver disease despite these patients being on immunosuppressants like CyA and MMF. So, it is difficult to know whether CyA and MMF per se have potent antiviral activity.

### 3.3. Glucocorticoids

Glucocorticoids are one of the key immunosuppressive drugs used in heart transplantation. Their immunosuppressive effect is mediated by modulating the genes that affect leucocyte function. Contrary to other immunosuppressants, glucocorticoid treatment has been shown to enhance viral replication. In patients with chronic HBV related hepatitis, glucocorticoid treatment [31] has been shown to increase levels of HBsAg, HBcAg, and HBV DNA in hepatocytes and also to increase the severity of liver disease and liver-related mortality. This occurs not only on high dose glucocorticoid treatment but also on low dose when administered in the long term [32]. Possible mechanisms suggested include increasing HBV DNA transcriptional activity and viremia via a corticosteroid responsive element in HBV genome [33]. 

## 4. Effect of Antiviral Therapy on Graft/Survival Outcome

HBV/HCV reactivation in HTX recipients has been shown to significantly affect posttransplantation survival. Prior to the introduction of antiviral therapy the reactivation rates in HBsAg +ve patients undergoing solid organ transplantation were over 50% [34]. The effect of these antiviral therapies in HTX recipients has been evaluated in a number of studies. 

Interferon-alpha has been traditionally avoided in HTX recipients due to the increased risk of acute cellular rejection. Interestingly, Fagiuoli et al. [35] observed no unexpected acute cellular rejection and major side effects when interferon-alpha was used in 7 HTX recipients (2 with HBV, 4 with HCV, and 1 with concomitant HBV/HCV infection). All patients in the HBV group showed complete and sustained virological response, whereas only 1 in the HCV group showed sustained response during the mean follow-up period of 8.5 ± 3 years. However, Wang et al. [36] reported a fatal outcome in a 50-year-old HTX recipient with coexistent HCV infection who was treated with peginterferon alpha-2b and ribavirin. The patient developed chronic hepatitis with elevated liver enzymes a year after HTX and was started on peginterferon alpha-2b and ribavirin. With six months of treatment, liver enzymes slowly returned towards the normal range and ribavirin was discontinued. The patient subsequently presented with severe heart failure and arrhythmias refractory to medical treatment. During autopsy, severe fatty degeneration of heart myocytes with patent coronary arteries and no evidence of rejection were noted which was felt to be confirmatory of peginterferon alpha-2b induced cardiotoxicity.

Lamivudine, a nucleotide analogue, has been widely used for HBV infection with successful results. However, emergence of resistant mutation over the years limits its use. It acts by causing premature termination of viral DNA chains during reverse transcription. In a retrospective study, Wang et al. [37] analysed the effect of lamivudine treatment of HBV infection in 14 HTX recipients. During follow-up, 4 patients died: 1 due to end-stage liver cirrhosis, 2 due to sudden death, and another due to diffuse B cell lymphoma 14–138 months after HTX. The patients who survived had normal liver enzymes and undetectable HBV-DNA levels but YMDD mutant occurred in 2 patients. The authors concluded that lamivudine treatment for HBV reactivation was safe and effective and may need to continue indefinitely unless a resistant mutation develops.

 In another pilot study, Potthoff et al. [38] assessed the efficacy of long-term antiviral therapy in 20 HTX recipients with chronic HBV infection. About 75% of patients had evidence of cirrhosis or bridging fibrosis at the start of treatment. Patients were initially treated with famciclovir and subsequently changed to lamivudine if they showed no response virologically. During the follow-up period of 103 months, only one patient was on famciclovir and 16 patients were switched to lamivudine after 0.5 to 4 years of famciclovir therapy. Of these, six patients showed long-term response to lamivudine therapy, whereas 10 patients (63%) developed resistance. Successful rescue therapy with adefovir (*n* = 3) and tenofovir (*n* = 1) was achieved in 4 of them with resistance. Nine patients died during follow-up and worryingly 5 of them were due to lamivudine-resistance-related liver failure.

## 5. Patient Survival/Graft Outcome in Other Solid Organ Transplantation Processes

### 5.1. Renal Transplantation

The prevalence of hepatitis B and hepatitis C infection in end-stage renal disease (ESRD) patients is over 10% [39, 40]. The success of renal transplantation (RTx) in this cohort depends mainly on the development of future complications like rejection, neoplasm, posttransplant diabetes, and glomerulonephritis. The effect of HBV/HCV infection on patient survival/graft outcome in RTx recipients has been evaluated in a number of studies (Table 2). 

In a retrospective analysis of 230 HCV infected patients, Roth et al. [15] assessed the long-term outcome of RTx in 110 patients. During follow-up, death from graft failure occurred in 15% of patients, whereas death due to other causes (with a functioning graft) occurred in 26% of patients. Death rate during the first 6 months after transplant was significantly higher as a result of infection. However, this risk was significantly lower beyond 6 months when compared with pretransplant. They also found that the liver histology remained stable or improved in 77% of RTx recipients (who underwent follow-up liver biopsies) and the 10-year patient and graft survival rate was 57 and 40%. The authors conclude that RTx confers a long-term survival benefit in this cohort when compared to those remaining on the waiting list. In another retrospective study, Knoll et al. [16] compared the outcomes in HCV+ RTx recipients and HCV+ ESRD patients who were on the waiting list. Of 2053 patients who were evaluated for RTx, 151 (7.4%) were found to be HCV+. Those patients who had at least 2 years follow-up were included. 33 patients received RTx and 25 were found to be acceptable but did not receive transplant during the follow-up. The results from this study showed that survival was significantly reduced in the HCV+ patients who were awaiting renal transplantation when compared to those who had transplant (*P* = 0.043). The authors of these studies suggested that ESRD patients should not deny RTx purely based on their HCV status.

 In a systematic review of six observational studies (6050 patients), Fabrizi et al. [17] examined the RTx outcomes in HBV+ recipients. The authors concluded that HBV+ RTx recipients are at increased risk for mortality and graft failure when compared with HBV− recipients. On the contrary, in a retrospective survey [18] of the Organ Procurement Transplant Network/United Network for Organ Sharing (OPTN/UNOS) registries, Reddy et al. found that the patient and graft survival in HBV+ RTx recipients was comparable to HBV− recipients. Of 75,861 recipients, 1346 were HBV+ (surface antigen positive) and 74,335 were HBV− (surface antigen negative). The 5-year patient survival rate was 85.3% in the former group and 85.6% in the latter group. Similarly, the graft survival rate at 5 years was 74.9% and 75.1% for HBV+ and HBV−, respectively. However, the cumulative incidence of liver failure was found to be increased in HBV+ compared to HBV− recipients (1.3% versus 0.2%; *P* < 0.001).

Though these studies were of retrospective nature, there appears to be a continuous improvement in survival outcome in this cohort over the years. This could be partly attributed to the introduction of new antiviral therapy. Given the higher mortality rate in ESRD patients who were on the waiting list and improved survival outcome after transplant, most of the authors recommend RTx in this cohort. 

### 5.2. Lung Transplantation

The survival/graft outcome of HCV infection/HbcAb+ allografts in lung transplant recipients was analysed in a limited number of studies. 

Sahi et al. [19] analysed the outcomes of HCV+ lung transplant recipients from the Cleveland Clinic Foundation's Lung Transplant database. Of 465 patients, six patients were identified to be HCV+. Evidence of cirrhosis was excluded by pretransplant liver biopsy in 5 patients. During the median follow-up period of 3.2 years, two patients died (one at 8 months and the other at 2 years), one developed bronchiolitis obliterans syndrome, and four were alive. Significant rise in HCV RNA levels without concomitant increase in transaminase levels was found after-transplant. No significant difference was found in patient or graft survival between the HCV+ and HCV− recipients from this study. 

In a retrospective analysis of 456 lung transplant recipients, Hartwig et al. [20] assessed the risk of viral transmission and safety in patients who received hepatitis B core antibody (HbcAb+) and hepatitis C antibody (HCV Ab+) pulmonary allografts. Twenty-nine patients (HB group) received HbcAb+ allografts and 3 (HC group) received HCV Ab+ allografts. The survival rate at 1 year was 83% in the HB group when compared to 82% who received non-HB organs. None of the patients in the HB group developed clinical viral disease due to viral hepatitis, whereas one died of liver failure in the HC group during follow-up. The authors conclude that the use of HbcAb+ pulmonary allografts in recipients with prior immunisation seems to be safe and effective strategy to overcome organ shortage. Similarly, analysis of 333 recipients of HbcAb+ donor organs from UNOS/OPTN registries had shown that donor HbcAb+ status did not impact 1- or 5-year survival after transplant. Authors conclude that lung and heart-lung allografts from HbcAb+ donors may be safely used and this would increase the number of transplants performed without compromising recipient outcomes [21].

 Although the above studies show encouraging results, they have several limitations including the retrospective nature, small cohort size, and short duration of follow-up. 

### 5.3. Liver Transplantation

Liver disease caused by HBV and HCV infection remains the main indication for liver transplantation in developed countries. Recurrence of HBV and HCV infection can pose a major problem after transplant and can lead to poor patient and graft survival. 

In a retrospective analysis of the UNOS registry, Singal et al. [22] analysed the frequency and outcomes of liver transplantation based on etiology of liver transplant. Between 1994 and 2009, 54,687 adult liver transplants were performed in the United States. Of these, 15,147 liver transplantation processes were done for HCV+ infection, 1816 were for HBV+, 6066 were for HCV+ and alcohol, and the rest were for other conditions causing chronic liver disease. The five-year graft and patient survival was 80–85% in HBV+ patients, whereas the HCV+, HCV+, and alcohol group had the worst outcome (hazard ratio, 1.5–2.4). This has been attributed to HCV recurrence with rapid progression of fibrosis resulting in cirrhosis. Interestingly, Reddy and Everson [23] reported on a 54-year-old man, who developed HCV recurrence after liver transplantation. Treatment with pegylated interferon and ribavirin resulted in rapid virological response but relapsed after 48 weeks of treatment. Patient was subsequently given a 48-week triple therapy regime when the new protease inhibitors (boceprevir) became available. HCV RNA levels were undetectable 12 weeks from the start of the therapy and the levels remained negative 12 weeks even after cessation of therapy. However, significant adverse effects and drug interactions were observed during the treatment period.

## 6. Summary

Hepatitis B or C infection is not an absolute contraindication for orthotopic heart transplantation. The National Health Service Blood and Transplant (NHSBT) organ donation statistics (2012-2013) clearly delineate the modest size of heart transplant program in UK compared to other organ transplantation processes: kidneys (*n* = 1750), liver (*n* = 775), lungs (*n* = 187), and heart (*n* = 142). Given the scarcity of donor organs and only limited evidence available on long-term survival outcome in this cohort, no clear recommendations can be made to list these patients for heart transplantation. The introduction of newer antiviral treatments in the treatment of hepatitis B and hepatitis C infection before and after transplantation seems promising but again their long-term effect is yet to be established. The hepatitis B drugs entecavir and tenofovir both have a high genetic barrier to the development of drug resistance but there is limited data available on their use in the cardiac transplant setting. There is currently no data available on the use of the new protease inhibitors for genotype I hepatitis C infection. Large multicentre randomised controlled trials with different antiviral therapies and immunosuppressive treatments are needed to resolve this issue and investigate the potential drug-drug interactions that may occur. Until then each case should be discussed on an individual basis.

## Figures and Tables

**Table 1:** Studies correlating HBV/HCV infection and clinical outcomes after cardiac transplantation.

Author	Country	Cohort size	Follow-up	Measure	Outcome
Haji et al., 2006 [10]	USA	*n* = 66 HBV group Donor HBcAb+ (*n* = 8) Recipient HBcAb+ (*n* = 5) Control group (*n* = 53)	1 year	HBV seropositivity with CAV	CAV risk increased when HBV seropositivity was found in either donor or recipient

Lee et al., 2011 [11]	USA	*n* = 20,687 HCV+ group (*n* = 443) HCV− group (*n* = 20,224)	5.6 years	HCV positivity (hepatitis C Ab +) and survival	Higher mortality in HCV+ group (most of the deaths due to CAV)

Fagiuoli et al., 2001 [12]	Italy	*n* = 49	8 ± 3.1 years	Prevalence, clinical course of HBV and HCV in HTX recipients	Significant proportion of patients with HBV and/or HCV infection developed chronic liver disease

Hosenpud et al., 2000 [13]	USA	*n* = 30	949 ± 598 days	Outcomes in patients who are HBsAg+ prior to HTX	Chronic liver disease is more common in HBsAg+ patients

HBV: hepatitis B virus, CAV: cardiac allograft vasculopathy, HBsAg: hepatitis B surface antigen, HCV: hepatitis C virus, HTX: heart transplantation, HBcAb: hepatitis B core antibody.

**Table 2:** Studies correlating HBV/HCV infection and clinical outcomes in other solid organ transplantation processes.

Author	Country	Transplanted organ	Cohort size	Follow-up	Measure	Outcome
Roth et al., 2011 [15]	USA	Kidney	*n* = 110	10 years	Long-term outcome of RTx in HCV+ patients	RTx confers a long-term survival benefit

Knoll et al., 1997 [16]	USA	Kidney	*n* = 2053 HCV+ *n* = 151 RTx *n* = 33 Waiting list *n* = 25	2 years	Outcomes in HCV+ RTx recipients to HCV+ ESRD patients	Decreased survival in HCV+ patients on waiting list compared to those who had RTx

Fabrizi et al., 2005 [17]	Italy	Kidney	*n* = 6050		Outcome of RTx in HBV+ patients	Increased mortality in HBV+ recipients than HBV− recipients

Reddy et al., 2011 [18]	USA	Kidney	*n* = 75,861 HBV+ *n* = 1346 HBV− *n* = 74,335	5 years	Patient/graft survival in HBV+ recipients	Patient/graft survival in HBV+ was comparable to HBV− recipients

Sahi et al., 2007 [19]	USA	Lung	*n* = 465 HCV+ *n* = 6	3.2 years	Outcome of lung transplant in HCV+ recipients compared to HCV− controls	No significant difference in patient and graft survival

Hartwig et al., 2005 [20]	USA	Lung	*n* = 456 HB group *n* = 29 HC group *n* = 3		Outcome of the use of HbcAb+ and HCVAb+ allografts	Use of HbcAb+ allografts in recipients with prior immunisation was safe

Dhillon et al., 2009 [21]	USA	Heart-lung	HbcAb+ *n* = 333 HbcAb− *n* = 13,233	5 years	Impact of donor HbcAb+ status on outcomes of lung and heart-lung recipients	Lungs and heart-lung allografts from HbcAb+ donors may be safely used

Singal et al., 2013 [22]	USA	Liver	*n* = 54687	5 years	Outcomes of liver transplant based on etiology of liver disease	Worst outcome in HCV+, HCV+, and alcohol

Reddy and Everson 2013 [23]	USA	Liver	*n* = 1	60 weeks	Treatment of HCV recurrence with protease inhibitor based therapy	Intervention with protease based therapy is justified in HCV eradication

HBV: hepatitis B virus, ESRD: end-stage renal disease, HbcAb: hepatitis B core antibody, HCV: hepatitis C virus, RTX: renal transplantation, HCV Ab: hepatitis C antibody.
